# To substitute or not? A systematic review of immunoglobulin replacement therapy in multiple myeloma patients treated with bispecific antibodies

**DOI:** 10.3389/fimmu.2025.1722579

**Published:** 2026-01-12

**Authors:** Juni Songe Paulsen, Tobias S. Slørdahl

**Affiliations:** 1 Department of Clinical and Molecular Medicine, Norwegian University of Science and Technology - NTNU, Trondheim, Norway; 2 Nordmøre og Romsdal Hospital (SNR), Møre og Romsdal Hospital Trust, Hjelset, Norway; 3 Department of Hematology, St. Olavs Hospital - Trondheim University Hospital, Trondheim, Norway

**Keywords:** BCMA-targeting bispecific antibodies, bispecific antibodies, GPRC5D-targeting bispecific antibodies, immunoglobulin replacement, immunoglobulin substitution, infections, IVIG, multiple myeloma

## Abstract

Bispecific antibodies are a novel class of immunotherapies that have demonstrated high response rates in heavily pretreated patients with multiple myeloma. However, their use is associated with increased risk of infections, which contribute to morbidity and mortality. Current guidelines recommend immunoglobulin replacement therapy for patients receiving bispecific antibodies with polyclonal IgG levels below 4 g/L, in addition to prophylactic antimicrobial therapy, to reduce infection risk. This systematic review aimed to evaluate the effect of immunoglobulin replacement therapy in multiple myeloma patients treated with bispecific antibodies. Through a structured literature search, we identified five retrospective, non-randomized cohort studies comprising a total of 653 patients. Three of these studies reported a significant reduction in infections, particularly severe infections, among patients receiving immunoglobulin replacement therapy. Given the substantial time and resource burden associated with continuous immunoglobulin prophylaxis for both patients and healthcare systems, further prospective, randomized studies are needed to confirm these findings and guide evidence-based practice.

## Introduction

Multiple myeloma (MM) is the second most common hematological malignancy, and prevalence is rising with an increasing incidence (1) and relative survival (2). Patients with multiple myeloma are considered immunosuppressed due to both the immunosuppressive nature of the disease and the effects of treatment. The disease disrupts normal B- and T-cell development and function, promotes an immunosuppressive bone marrow microenvironment, and leads to hypogammaglobulinemia. Additionally, therapeutic agents such as proteasome inhibitors, anti-CD38 monoclonal antibodies, immunomodulatory drugs (IMiDs), and corticosteroids further contribute to immunosuppression (3). The immunosuppressive state of MM is reflected through a markedly increased risk of infections compared to matched controls (4). Guidelines on infection prophylaxis in MM therefore recommend antimicrobial prophylaxis with antivirals, antibiotics, and immunoglobulin replacement therapy (IgRT) based on treatment administered, the grade of hypogammaglobulinemia, and the presence of recurrent infection (5).

In recent years, a range of new medications have been introduced as standard-of-care treatment of MM, including immunotherapies such as bispecific antibodies (BsAbs) (6–9). BsAbs target both T cells and myeloma cells, leading to T-cell activation and tumor killing. They are given as continuous treatment after an initial step-up dosing. U.S. Food and Drug Administration (FDA)- and European Medicines Agency (EMA)- approved BsAbs include linvoseltamab, teclistamab, and elranatamab targeting B-cell maturation antigen (BCMA) and talquetamab targeting G-coupled protein receptor, class C, group 5, member D (GPRC5D) on myeloma cells, showing high responses in heavily pretreated patients. Toxicities include cytokine release syndrome (CRS) and immune effector cell-associated neurotoxicity syndrome (ICANS), typically occurring with initial doses. In addition, infections have been recognized as a major side effect (10). Following treatment with BsAbs, more than half of patients develop an infection, with 24% experiencing grade 3 or higher infections. The risk of serious infection is greatest in those treated with BCMA-targeted BsAbs and in patients receiving BsAbs in combination with other anti-myeloma therapies (11). Types of infections vary and include bacterial, viral, and fungal infections, such as the opportunistic infections *Pneumocystis jirovecii* and cytomegalovirus (CMV) reactivation (10). The most common site of infection is the respiratory tract (12).

One of the factors contributing to the increased infection risk in patients treated with BsAbs is the high rate of hypogammaglobulinemia (74.5%–87.0%) (6–8, 13). Current recommendations advise IgRT when polyclonal IgG levels fall below 4.0 g/L (14). IgRT is usually given as intravenous immunoglobulin (IVIG) infusions or as subcutaneous immunoglobulin (SCIG) infusions within healthcare facilities. As BsAb use grows in MM, severe hypogammaglobulinemia (IgG < 4g/L) is expected to become more prevalent among MM patients. Consequently, current treatment guidelines are likely to drive greater utilization of IgRT. This contributes to a greater strain on the healthcare system, leading to increased costs and to elevated time toxicity for patients. Therefore, research on the effect of IgRT in this setting is important for optimizing patient care. IgRT may reduce symptom burden and facilitate the continuation of uninterrupted treatment with BsAbs. Robust evidence supporting the effect of IgRT as primary or secondary prophylaxis is limited, with few studies and no randomized controlled trials available. Furthermore, some findings have been contradictory, reflecting heterogeneity in study design and patient populations. To address this gap, we conducted a comprehensive search of all available literature to identify relevant evidence. The aim of this review was to evaluate the impact of IgRT in multiple myeloma patients receiving bispecific antibodies.

## Methods

The search was conducted in PubMed/Medline and Embase. Using thesaurus and the relevant free-text terms, the following three main concepts were used: “multiple myeloma”, “bispecific antibodies”, and “immunoglobulin replacement therapy”. For the bispecific antibodies, the following names of the four drugs approved by the FDA and EMA were included: linvoseltamab, teclistamab, talquetamab, and elranatamab. The concepts were combined using the Boolean operator OR, and at the end, every main concept was combined using AND. The search was performed on June 15, 2025. No restriction on publication date was applied. A detailed description of search strategies applied in the two databases is provided in **Supplementary Table S1**.

Both authors independently reviewed the records. Records found by searching the combined concepts were first screened by title for relevance to the review question. The relevant records were then screened by reading abstracts or searching for keywords. Inclusion criteria comprised English-language primary research studies and treatments involving bispecific antibodies targeting BCMA or GPRC5D. For comparability, the effect on rates of infection was focused on, incorporating this as an inclusion criterion. Articles were excluded if they did not evaluate the effect of immunoglobulin replacement on infections; or if they were case reports, reviews, consensus recommendations, or updates; or if they were published in languages other than English. The remaining articles were fully reviewed to assess relevance.

The studies were evaluated using the Critical Appraisal Skills Program checklist for cohort studies (15). The first section evaluated the validity of the study by assessing the focus of the study, cohort recruitment, the measurement of exposure and outcomes, the evaluation of confounding factors, and follow-up. The second section assessed the precision and reliability of the results. The final section evaluated transferability, reproducibility, and the implications of the results.

## Results

**Figure 1:**
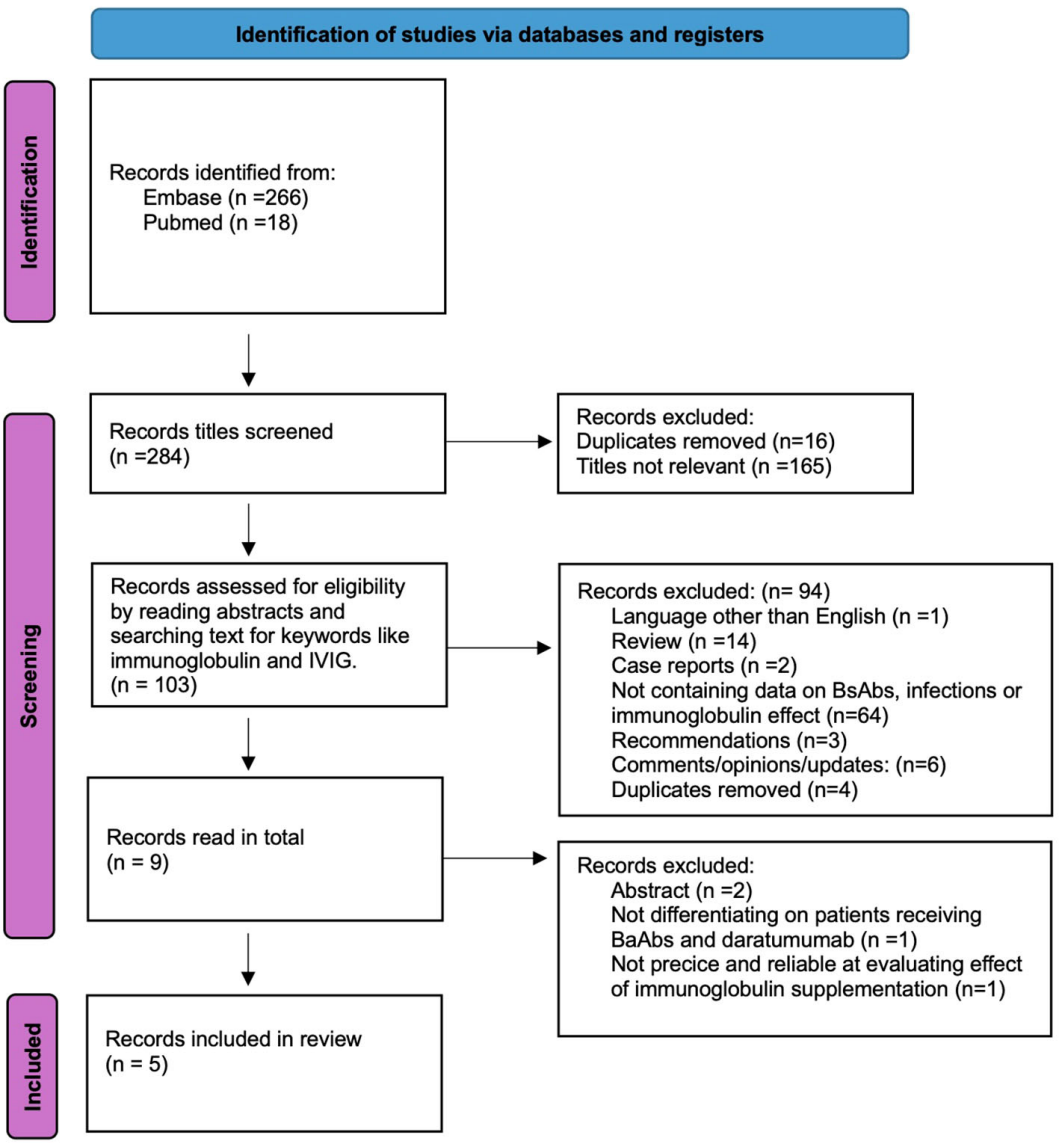
PRISMA flow diagram for systematic review.

The search combining these concepts resulted in 18 records in PubMed/Medline and 266 records in Embase. The titles were first screened for relevance, excluding 181 records. The remaining 103 records were screened by reading abstracts or searching for keywords. This process yielded nine records that were read in total. Four records were subsequently excluded: two, as they were conference abstracts reporting the same data as journal articles included; one, as it evaluated the effect of IVIG in a cohort of patients receiving either daratumumab or BsAbs; and one, as it evaluated infections in patients receiving prophylactic IgRT without any control group for comparison. The process is described in the Preferred Reporting Items for Systematic reviews and Meta-Analyses (PRISMA) flow diagram in **Figure 1**. All five remaining records included in the review were retrospective, non-randomized cohort studies. They are summarized in **Table 1**. **Table 2** contains a summary of patient characteristics in each study. Of the records included, all were journal articles. The records were evaluated using the CASP Cohort study checklist, shown in **Supplementary Table S2**.

**Table 1:** Summarized details of included records.

Author, year, title, journal, location	Purpose	# of patients	Subject characteristics	Study design	Instruments used	Time period	Main findings and comments
Kristine A. Frerichs et al., 2024, Teclistamab impairs humoral immunity in patients with heavily pretreated myeloma: importance of immunoglobulin supplementation, *Blood Advances*, The Netherlands (13)	To improve understanding of how teclistamab impacts humoral immunity, effect of infectious prophylaxis, and vaccination strategy.	165 (52 evaluated for effect of IVIG)	Patients with relapsed/refractory MM, ECOG 0–1, who had received 3 or more prior lines of therapy, including a proteasome inhibitor, an immunomodulatory drug, and an anti-CD38 monoclonal antibody. Patients who had received prior BCMA-targeted therapy were excluded.	Retrospective cohort study in patients receiving the pivotal recommended phase 2 dose from the MajesTEC-1 study: an open-label, multicenter phase 1 and phase 2 study	Data collection on baseline characteristics, infections, vaccination, vaccine responses, drug administration, and serum measurements at different time points.	From March 3, 2020, to data cut-off on June 1, 2023	Teclistamab caused reduction in polyclonal immunoglobulin levels, normal plasma cell depletion, B-cell reduction, and impaired humoral immune response after vaccination. IVIG supplementation caused significant reduction in serious infections.
Guido Lanceman et al., 2023, IVIg use associated with ten-fold reduction of serious infections in multiple myeloma patients treated with Anti-BCMA Bispecific antibodies, *Blood Cancer Discovery*, USA (16)	Describing infections and their risk factors. Evaluating impact of infection prophylaxis in patients treated with BCMA-targeted BsAbs.	37	All multiple myeloma patients treated with at least 1 dose of BCMA-targeting BsAbs at Mount Sinai Hospital in New York in four different clinical trials.	Retrospective cohort study including patients from four clinical trials.	Data collection by retrospective review on baseline characteristics, treatment, disease response, hypogammaglobulinemia, infections, and infection prophylaxis.	January 1, 2019, to June 30, 2022.	Profound hypogammaglobulinemia was universal in patients responding to bispecific antibodies. Patients had increased risks of infection. Risk of grade 3–5 infections was lower when using IVIG.
Aurélie Jourdes et al., 2024, “Characteristics and incidence of infections in patients with multiple myeloma treated by bispecific antibodies: a national retrospective study”, *Clinical Microbiology and Infection*, France (12)	Exploring real-world data on epidemiology, characteristics, risk factors, and outcomes of infections in patients treated with BsAbs.	229	All patients with multiple myeloma receiving bispecific antibodies (200 BCMA-targeting and 29 GPRC5d-targeting) in 14 French centers.	Multicenter retrospective cohort study	Data collection on patient characteristics, infections, hypogammaglobulinemia, corticosteroid, and immunoglobulin substitution.	December 2020 to February 2023.	Bispecific antibodies were associated with frequent infections affecting management of patients. An association between corticosteroid use during ICANS or CRS and higher risk of first infection was found. No association between immunoglobulin substitution and infection risk.
Meera Mohan et al., 2024, “Teclistamab in relapsed refractory multiple myeloma: multi-institutional real-world study”, *Blood Cancer Journal*, USA (17)	To explore safety and efficacy of teclistamab in the real-world setting in patients with relapsed/refractory multiple myeloma.	110	Including all patients who received one dose or more of teclistamab across 5 academic centers in the USA.	Multicenter retrospective cohort study	Data collection on patient demographics, disease characteristics, baseline advanced imaging, and baseline and serial immunoglobulin levels. Infections were confirmed by clinical, imaging, microbiological, or histopathological evidence from day 1 until 60 days after completion of therapy.	Patients receiving teclistamab between January 2023 and August 2023, including 60 days after last dose.	Proportion of patients who achieved VGPR or better was comparable to results from the MajesTEC-1 trial. The incidence of CRS and ICANS was higher than in the MajesTEC-1 trial, although it remained low. The incidence of infections was lower. Primary immunoglobulin prophylaxis was associated with a significantly lower incidence of infections.
Meera Mohan et al., 2025, “Effect of Intravenous Immunoglobulin (IVIG) Supplementation on infection-free survival in recipients of BCMA-directed bispecific antibody therapy for multiple myeloma”, *Blood Cancer Journal*, USA (18)	Evaluating the effect of immunoglobulin substitution as primary prophylaxis in patients receiving BCMA-directed bispecific antibodies.	225	Patients receiving teclistamab as standard of care or alternative BCMA targeting bispecific antibodies at 5 academic centers in the USA.	Multi-institutional retrospective cohort study	Data collection on baseline characteristics, disease characteristics, infections (confirmed by imaging, histopathological, microbiological, or clinical tests), and survival	Patients receiving BCMA-targeting bispecific antibodies between Nov 2017 and Dec 2023.	Primary prophylaxis with IVIG is significantly associated with a reduction in infection-free survival and with improved overall survival. Tocilizumab was linked to increased risk of infections.

ECOG, Eastern Cooperative Oncology Group (performance status scale); BsAbs, bispecific antibodies; BCMA, B-cell maturation antigen; GPRC5D, G-coupled protein receptor, class C, group 5, member D; ICANS, immune effector cell-associated neurotoxicity; CRS, cytokine release syndrome; IVIG, intravenous immunoglobulin; MM, multiple myeloma.

**Table 2:** Summarized characteristics of participants in each study.

Record	Frerichs et al., 2024 (13)	Lancman et al. (16)	Jourdes et al., 2024 (12)	Mohan et al., 2024 (17)	Mohan et al., 2025 (18)
Number of patients	52 of 165	37 patients were on IVIG 56% of the time.	229	110	225
pIVIG prophylaxis	20 (38%)	NR	NR	46 (43%)	92 (41%)
Non-pIVIG prophylaxis	32 (62%)	NR	NR	64 (57%)	133 (59%)
Male/female	56%/44% non-pIVIG 55%/45% pIVIG	38%/62%	51%/49%	51%/49%	53%/47% non-pIVIG 50%/50% pIVIG
Median age	64	66	67	68	69 (pIVIG) 71 (non-pIVIG)
Median number of previous lines	5 in pIVIG 6 in non-pIVIG	7	4	6	5 in non-pIVIG 5 in pIVIG
Triple-class refractory^†^	77.6%	78%	82%	86%	92% in non-pIVIG 79% in pIVIG
Penta-drug refractory^††^	15% in pIVIG 31% in non-pIVIG	24%	NR	76%	NR
Previous treatment with bispecific antibodies or CAR-T	5% in pIVIG 9% in non-pIVIG	32%	NR	35%	Previous BCMA-directed therapy: 20% in non-pIVIG 27% in pIVIG
Infections	1.36 per patient-year in non-pIVIG group, 0.12 per patient-year in pIVIG group	Median 2 (0-10)	Median 1 (1-7)	78 infections in 44 patients	288 infections in 136 patients
Patients in non-pIVIG started on secondary IVIG prophylaxis (%)	14 (77%)	NA	NR	NR	39 (29%)

BCMA, B-cell maturation antigen; GPRC5D, G-coupled protein receptor, class C, group 5, member D; CAR-T, chimeric antigen receptor T-cell; IVIG, intravenous immunoglobulins; pIVIG, primary prophylactic intravenous immunoglobulins; NR, not reported; NA, not applicable.

^†^Triple-class refractory: defined as refractory to at least one proteasome inhibitor, immunomodulatory drug, and anti-CD38 antibody.

^††^Penta-drug refractory: defined as refractory to lenalidomide, pomalidomide, bortezomib, carfilzomib, and anti-CD38-antibody.

Frerichs et al. (13) studied the effect of IgRT in a cohort (n = 52) of relapsed refractory (RR) MM patients receiving teclistamab in the MajesTEC-1 trial (6). The study found a rapid reduction of polyclonal immunoglobulin after initiating teclistamab. They also found severely impaired vaccine responses to *Streptococcus pneumoniae*, *Haemophilus influenzae*, and *SARS-CoV-2* vaccines in a subset of the patients. IgRT was used either as primary prophylaxis in patients with polyclonal IgG of <4 g/L (pIVIG group) or as secondary prophylaxis in patients with severe infection and IgG of <4 g/L (non-pIVIG group), as per the physician’s discretion. IgRT as primary prophylaxis was associated with a significantly lower risk of infection with an incidence rate of 0.12 per patient-year in the group receiving pIVIG versus 1.36 per patient-year in the non-pIVIG group; 10% and 56% of patients experienced ≥grade 3 infections in the pIVIG and non-pIVIG groups, respectively. A similar cohort study of clinical trial patients was conducted by Lancman et al. (16). They retrospectively reviewed charts of heavily pretreated RR MM patients (n = 37) receiving BCMA-targeting bispecific antibodies. Infections were classified as occurring during periods ON or OFF IgRT, indicating that patients could belong to both the ON-IVIG and OFF-IVIG groups at different times. Patients were on IgRT 58% of the time. There was a 90% lower rate of grade 3–5 infections when patients were receiving IVIG, but no significant difference in grade 1–5 infections or in grade 3–5 bacterial infections. They also reported that the cumulative probability of infections increased over time and that most infections occurred during disease remission.

The other three studies were either true real-world studies (17), studies with mixed real-world and clinical trial patients (18), or not specified (12). Jourdes et al. (12) conducted a national, multicenter study in France that included 229 patients across 14 centers to assess infections that affected patient management, defined as those resulting in hospitalization, necessitating specific treatment, or requiring modification of BsAb administration. The study included patients receiving either GPRC5D-targeting (13%) or BCMA-targeting (87%) BsAbs. Exposure to immunoglobulin supplementation was not included in the record. They reported 234 infections, of which 123 (53%) were ≥grade 3. In exploratory univariate analyses, immunoglobulin levels <4 g/L and IgRT were not significantly associated with a lower risk of infections. In the first paper of Mohan et al. (17), they included 110 patients receiving at least one dose of teclistamab at five US centers, evaluating the safety and efficacy of the drug as standard of care. Intravenous immunoglobulins were given as primary prophylaxis in 43% of patients with hypogammaglobulinemia, regardless of infection history. The cumulative incidence of infections in patients on primary IVIG prophylaxis (pIVIG) was 35% and 35% at 3 and 6 months, compared to 44% and 54% in patients not on pIVIG, respectively. The difference was even greater for grade 3–5 infections, but for all-grade and grade 3–5 infections, confidence intervals were overlapping. Patients on pIVIG had a statistically significant reduction in infections, with a relative risk reduction of approximately 70%. The second record from Mohan et al. (18) from 2025 evaluated the effect of IgRT on clinical outcomes in patients receiving BCMA-targeted BsAbs. Patients on IVIG prior to the start of bispecific antibodies were excluded. The authors could not find a significant difference in the cumulative incidence at 12 months of all-grade (56% *vs*. 60%) and ≥3 grade infections (35% *vs*. 45%) between the pIVIG and non-pIVIG groups. They did, however, find a significantly better infection-free survival (IFS) in the pIVIG group, 7.7 *vs*. 3.0 months. Patients receiving pIVIG had superior median overall survival (44 *vs*. 16 months). In a multivariate analysis, they could not find that primary IVIG prophylaxis had a significant protective effect against infections (HR = 0.71), but secondary IVIG prophylaxis did (HR = 0.41).

In four of the studies, analyses were performed to identify other risk factors that influenced the likelihood of infection. In multivariate analyses, baseline lymphopenia (HR = 1.34) (18), administration of tocilizumab (HR = 1.53) (18), administration of corticosteroid for CRS/ICANS (HR = 2.01) (12), higher number of median prior lines of therapy (HR = 1.08) (18), and history of prior infection while on BsAbs (HR = 1.25–1.39) (17, 18) increased the risk of infections.

## Discussion

This systematic review critically evaluates all published studies of immunoglobulin replacement therapy on the risk of infections in MM patients treated with bispecific antibodies. None of the studies included were randomized, and all of them were retrospective cohort studies, making the results to some degree uncertain. In three of the five studies (13, 16, 17) included, a significant effect of immunoglobulin replacement therapy on the risk of infections was reported, one of them only in reducing grade 3–5 infections (16). Two studies did not find a significant decrease in infections in patients receiving IgRT (12, 18). Lack of risk reduction in two of the trials could have multiple explanations. In the large French real-world study, the authors included both patients treated with GPRC5D- and BCMA-targeting BsAbs. GPRC5D-targeting bispecific antibodies are associated with lower infection rates compared to BCMA-targeting bispecific antibodies, potentially due to minimal GPRC5D expression on normal immune cells, which reduces immune-related toxicity (10, 19). The study also had heterogeneous use of other antimicrobial prophylaxis, and the record did not report exposure to immunoglobulin supplementation, making the results less reliable. The other study, not showing a significant reduction in the incidence of infection, may be related to a low number of patients receiving primary IVIG prophylaxis in the study and possibly due to the short follow-up (18).

Two of the articles included patients from clinical studies (13, 16). The other three studies were true real-world studies (17), studies with mixed real-world and clinical trial patients (18), or not specified (12), reflecting more the actual population and reporting of infections. Clinical trial populations often do not represent real-world myeloma patients, as many fail to meet trial inclusion criteria, limiting generalizability (20). The two largest real-world studies in this review could not find a significant difference in infection risk (12, 18).

Two of the studies from Mohan likely contained patients from the same population, which potentially introduced bias in the results, including overlap bias, increasing the weight of that cohort in effect estimates (17, 18). In the initial article from Mohan, a significantly lower infection risk was observed in patients receiving primary prophylaxis with immunoglobulins. However, in the subsequent study, the inclusion window was longer with patients from the same inclusion sites. The larger cohort showed no significant difference in the cumulative incidence of infections at 12 months, suggesting a smaller or non-significant effect. It did, however, find significantly longer infection-free survival and overall survival in the patients receiving IVIG. The findings are difficult to interpret due to baseline imbalances between groups. The non-pIVIG cohort had a higher proportion of patients with extramedullary disease (47% *vs*. 28%) and a greater percentage of triple-class refractory patients (92% *vs*. 79%). Additionally, patients in the non-pIVIG group received a median of only seven doses over 2 months of BsAb treatment, potentially indicating refractoriness to BsAbs that led to early treatment discontinuation. Had a prolonged infection-free survival been demonstrated in a prospective, randomized trial, the findings would have had substantial implications for clinical practice, supporting the use of immunoglobulin replacement as primary prophylaxis. In such a scenario, the additional time commitment, costs, and treatment burden associated with immunoglobulin administration would likely be justified by the benefit of extended survival and potentially increased quality of life.

Assessing the effect of IgRT was not among the primary objectives in all the studies included in this review; thus, reporting on IgRT exposure or reasons for starting IgRT was limited in three of the records. Study designs and patient group comparisons were inconsistent. Three studies evaluated primary versus secondary prophylaxis (13, 17, 18), while two others simply compared immunoglobulin replacement to no replacement (12, 16). Immunoglobulin use was heterogeneous and largely determined by physician discretion. Four studies also had heterogeneous use of infectious prophylaxis, such as vaccines and antibiotics, which could affect infection risk. There was a lack of adjustment for confounding factors affecting infection risk—factors that may lead the physician to choose prophylactic immunoglobulin substitution—potentially biasing results. The large number of possible causes increasing the risk of infection—including age (21), history of infections, lymphopenia (19), use of antimicrobial prophylaxis, and vaccine status—makes addressing confounding factors difficult. In addition, retrospective infection reporting is challenging due to variability in how cases are documented and the involvement of multiple healthcare institutions, which can result in inconsistent and incomplete data. This impacts the validity of infections as a measure of the effect of immunoglobulin supplementation.

Immunoglobulin replacement entails substantial costs, driven largely by plasma acquisition and manufacturing complexities. Costs vary with administration route, with potential savings achieved through patient training for self-administered subcutaneous immunoglobulins, which reduces the need for hospital-based intravenous infusions. However, SCIG infusions disrupt daily activities due to frequent self-administration, while intravenous immunoglobulins require more regular hospital visits.

Immunoglobulin replacement has been important in the supportive care of MM for decades. When to initiate IgRT has, however, been a topic of discussion based on the limited research data and conflicting results. A randomized, placebo-controlled, double-blind study with myeloma patients in the plateau phase in 1994 found significantly lower rates of infections in patients on IVIG (19 in 449 patient-months) compared to patients receiving placebo (38 in 470 patient-months) (22). Another randomized trial by Vacca et al. (23) randomized 46 MM patients with hypogammaglobulinemia (IgG < 5 g/L) to either SCIG infusions or observation. The number of infections was significantly lower in the SCIG group, compared to controls, including both all-grade infections and severe infections. Blombery et al. (24) retrospectively studied the effect of prophylactic IVIG in 266 MM patients undergoing autologous stem cell transplantation (ASCT) and could not find a significant difference in infectious complications between those who received prophylactic IVIG and those who did not. A large real-world retrospective study by O’Donnell et al. (n = 6062) compared the incidence of infections before and after IgRT initiation in patients treated with one or more doses of IgRT (25). There were significantly lower odds of infections after initiation of IgRT. This study likely focused primarily on the use of IgRT as secondary prophylaxis, as 67.0% of patients in the trial had hypogammaglobulinemia and only 7.8% of patients received IgRT. The study also showed a decreased use of antimicrobials in patients on IgRT. A multicenter real-world study from Australia, New Zealand, Korea, and Singapore (n = 2,374: 7.1% received immunoglobulin replacement therapy) found no overall survival benefit associated with IgRT use in myeloma patients (26). Based on these studies, the current consensus among patients on conventional myeloma therapies has therefore been to reserve IgRT for patients with hypogammaglobulinemia and recurrent serious infections (27).

Patients receiving bispecific antibodies are subjected to higher rates of infections (10), with some even leading to death in patients who are in complete remission (16). As hypogammaglobulinemia may contribute to the increased infection risk (28), it is important to consider treatment. According to Lancman et al. (16), hypogammaglobulinemia is universal in patients responding to BCMA-targeting BsAbs. Some risk factors for infection, other than hypogammaglobulinemia and IgRT, were identified in the studies, including administration of tocilizumab and steroids for CRS/ICANS, a higher number of prior lines of therapy, baseline lymphopenia, and a history of infection while on BsAbs. Larger datasets are likely required to more accurately identify significant risk factors. Still, there are no randomized trials, and the studies we found were characterized by uncertainties, biases, differences in populations compared, overlapping confidence intervals, and disparities in statistical significance of the results. In addition, evidence of benefit for broader clinical endpoints (e.g., survival) in the modern era is mixed. Since continuous prophylactic immunoglobulin substitution carries substantial implications, further increasing the already considerable time and resource demands placed on both patients and the healthcare system, prospective, randomized trials are warranted. Future studies should assess the optimal IgG threshold, dosing strategies, treatment duration, and which patients derive the greatest benefit from IgRT.

Soon to start enrollment is the TAK339 study (NCT06980480), including patients receiving teclistamab. This is a prospective, randomized controlled study in which the primary prevention group will receive immunoglobulin replacement for 12 months, while the secondary prevention group will receive immunoglobulin replacement only after at least one serious infection. To our knowledge, this is the only planned or ongoing randomized study on the effect of IVIG in patients receiving BsAbs, and the results will indicate whether IgRT confers benefit and should be used as primary or secondary prophylaxis. The trial may also validate IgG level monitoring and dosing intervals, thereby informing clinical guidelines for bispecific antibody treatment. There is a need to assess the proper cut-off IgG level in these patients. An ongoing prospective, randomized clinical trial aims to answer this in the setting of secondary IVIG prophylaxis in patients on BsAbs targeting BCMA (NCT07094048). Lastly, we need to determine if other ways of administering BsAbs may reduce the risk of infections without sacrificing effectiveness. The ongoing US “Limited-duration Teclistamab” trial (NCT05932680) looks at treatment discontinuation in patients on teclistamab achieving ≥ Very Good Partial Response (VGPR) after 6–9 months of therapy, measuring the rate of infectious complications 12 months after treatment discontinuation and immunoglobulin levels 2 years after discontinuation. Larger ongoing prospective real-world studies may also contribute to better answers in the future, including the population-based Norwegian Immunotherapy in Multiple Myeloma Study (NCT06855121), where all infections grade 1–5 and all use of infectious prophylaxis (including IgRT) are registered prospectively.

## Conclusion

In this systematic review examining the impact of immunoglobulin replacement therapy in patients with multiple myeloma receiving treatment with bispecific antibodies, three out of five studies found a statistically significant reduction in infections in patients treated with immunoglobulin replacement. The reduction was most pronounced for grade 3–5 infections. The two trials that did not show a significant reduction in infections, nevertheless, demonstrated the same trend. In addition to their retrospective and non−randomized design, heterogeneity in study design, differences in patient populations, variation in immunoglobulin replacement exposure, inadequate handling of biases, and lack of adjustment for confounding factors limit the certainty of these findings. Because IgRT is a blood product that requires continuous, time- and cost−intensive administration once initiated, it is essential to ensure that treatment is delivered to the right patients at the most appropriate time in their disease course. Further prospective, randomized studies are needed to guide treatment decisions. While awaiting randomized controlled trial data, this review suggests that patients receiving BCMA−targeted BsAbs who have hypogammaglobulinemia (IgG < 4 g/L) should be recommended prophylactic immunoglobulin replacement therapy or encouraged to participate in IgRT clinical trials.

## Data Availability

The original contributions presented in the study are included in the article/**Supplementary Material**. Further inquiries can be directed to the corresponding author.
