# Supplementary material for: To substitute or not? A systematic review of immunoglobulin replacement therapy in multiple myeloma patients treated with bispecific antibodies
Source: Front Immunol. 2026 Jan 12;16:1722579. doi: 10.3389/fimmu.2025.1722579 (PMC12832799; doi:10.3389/fimmu.2025.1722579)
Supplement: Supplementary file 1 [file Table1.docx]

**To substitute or not? A systematic review on the effect of immunoglobulin replacement in patients with Multiple myeloma receiving bispecific antibodies.**

Juni Songe Paulsen^1,2,3^ and Tobias S. Slørdahl^1,3^

^1^Department of Clinical and Molecular Medicine, Norwegian University of Science and Technology - NTNU, Trondheim, Norway

^2^Nordmøre og Romsdal Hospital (SNR), Møre og Romsdal Hospital trust, Hjelset, Norway

^3^Department of Hematology, St. Olavs hospital - Trondheim University Hospital, Trondheim, Norway

**SUPPLEMENTARY TABLES**

**Supplementary table 1: Search details**

PubMed

2025/06/15

| **Row** | **Query** | **Results** |
| --- | --- | --- |
| 1 | multiple myeloma[MeSH Terms] | 50,944 |
| 2 | "Multiple myeloma" | 66,573 |
| 3 | Myeloma | 82,456 |
| 4 | "Plasma cell myeloma" | 986 |
| 5 | "Plasmacytoma" | 11,652 |
| 6 | **((((multiple myeloma[MeSH Terms]) OR ("Multiple myeloma")) OR (Myeloma)) OR ("Plasma cell myeloma")) OR ("Plasmacytoma")** | 89,517 |
| 7 | bispecific antibodies[MeSH Terms] | 4,881 |
| 8 | "Bi-specific antibodies"[title/abstract:~2] | 162 |
| 9 | "Bispecific antibodies"[title/abstract:~2] | 3,520 |
| 10 | "Bi-specific antibody"[title/abstract:~2] | 152 |
| 11 | "Bispecific antibody"[title/abstract:~2] | 3,779 |
| 12 | "BsMab*" | 116 |
| 13 | "BsAb*" | 1,807 |
| 14 | "Bispecific T-cell engager" | 638 |
| 15 | "Bi-specific T-cell engager*" | 81 |
| 16 | Teclistamab | 171 |
| 17 | Talquetamab | 79 |
| 18 | Elranatamab | 65 |
| 19 | Linvoseltamab | 8 |
| 20 | Biab | 81 |
| 21 | Biabs | 55 |
| 22 | (((((((((((((bispecific antibodies[MeSH Terms]) OR ("Bi-specific antibodies"[title/abstract:~2])) OR ("Bispecific antibodies"[title/abstract:~2])) OR ("Bi-specific antibody"[title/abstract:~2])) OR ("Bispecific antibody"[title/abstract:~2])) OR ("BsMab*")) OR ("BsAb*")) OR ("Bispecific T-cell engager")) OR ("Bi-specific T-cell engager*")) OR (Teclistamab)) OR (Talquetamab)) OR (Elranatamab)) OR (Linvoseltamab)) OR (Biabs)) OR (Biab) | 8,885 |
| 23 | intravenous immunoglobulin[MeSH Terms] | 16,446 |
| 24 | "Immunoglobulin replacement" | 977 |
| 25 | "Immunoglobulin replacement therapy" | 673 |
| 26 | "Intravenous immunoglobulin" | 15,779 |
| 27 | IVIG | 24,166 |
| 28 | SCIG | 451 |
| 29 | "Subcutaneous immunoglobulin*" | 596 |
| 30 | "Immunoglobulin therapy"[title/abstract:~2] | 4,522 |
| 31 | "Immunoglobulin therapies"[title/abstract:~2] | 200 |
| 32 | "IgG RT" | 6 |
| 33 | "IgG replacement" | 136 |
| 34 | (((((((((intravenous immunoglobulin[MeSH Terms]) OR ("Immunoglobulin replacement")) OR ("Immunoglobulin replacement therapy")) OR ("Intravenous immunoglobulin")) OR (IVIG)) OR (SCIG)) OR ("Subcutaneous immunoglobulin*")) OR ("Immunoglobulin therapy"[title/abstract:~2])) OR ("Immunoglobulin therapies"[title/abstract:~2])) OR ("IgG RT")) OR ("IgG replacement") | 30,980 |
| 35 | ((((((multiple myeloma[MeSH Terms]) OR ("Multiple myeloma")) OR (Myeloma)) OR ("Plasma cell myeloma")) OR ("Plasmacytoma")) AND (((((((((((((((bispecific antibodies[MeSH Terms]) OR ("Bi-specific antibodies"[title/abstract:~2])) OR ("Bispecific antibodies"[title/abstract:~2])) OR ("Bi-specific antibody"[title/abstract:~2])) OR ("Bispecific antibody"[title/abstract:~2]))  OR ("BsMab*")) OR ("BsAb*")) OR ("Bispecific T-cell engager")) OR ("Bi-specific T-cell engager*")) OR (Teclistamab)) OR (Talquetamab)) OR (Elranatamab)) OR (Linvoseltamab)) OR (Biabs)) OR (Biab))) AND (((((((((((intravenous immunoglobulin[MeSH Terms]) OR ("Immunoglobulin replacement")) OR ("Immunoglobulin replacement therapy")) OR ("Intravenous immunoglobulin")) OR (IVIG)) OR (SCIG)) OR ("Subcutaneous immunoglobulin*")) OR ("Immunoglobulin therapy"[title/abstract:~2])) OR ("Immunoglobulin therapies"[title/abstract:~2])) OR ("IgG RT")) OR ("IgG replacement")) | 18 |

Embase

2025/06/15

| **Row** | **Query** | **Results** |
| --- | --- | --- |
| 1 | 'multiple myeloma'/exp | 118,333 |
| 2 | 'multiple myeloma':ti,ab,kw | 96,243 |
| 3 | 'myeloma':ti,ab,kw | 118,822 |
| 4 | 'plasma cell myeloma':ti,ab,kw | 1,870 |
| 5 | 'plasmacytoma':ti,ab,kw | 8,774 |
| 6 | #1 OR #2 OR #3 OR #4 OR #5 | 149,410 |
| 7 | 'bispecific antibody'/exp | 8,819 |
| 8 | 'bispecific t cell engager'/exp | 116 |
| 9 | ('bi specific' NEAR/3 antibodies):ab,kw,ti | 251 |
| 10 | (bispecific NEAR/3 antibodies):ab,kw,ti | 5,334 |
| 11 | ('bi specific' NEAR/3 antibody):ab,kw,ti | 353 |
| 12 | (bispecific NEAR/3 antibody):ab,kw,ti | 7,931 |
| 13 | bsmab*:ab,kw,ti | 142 |
| 14 | biabs:ab,kw,ti | 91 |
| 15 | biab:ab,kw,ti | 149 |
| 16 | bsab:ab,kw,ti | 1,411 |
| 17 | bi-specific t-cell engager*:ab,kw,ti | 199 |
| 18 | bispecific t-cell engager*:ab,kw,ti | 1,856 |
| 19 | teclistamab:ab,kw,ti | 423 |
| 20 | talquetamab:ab,kw,ti | 209 |
| 21 | elranatamab:ab,kw,ti | 193 |
| 22 | linvoseltamab:ab,kw,ti | 33 |
| 23 | #7 OR #8 OR #9 OR #10 OR #11 OR #12 OR #13 OR #14 OR #15 OR #16 OR #17 OR #18 OR #19 OR #20 OR #21 OR #22 | 14,363 |
| 24 | 'immunoglobulin'/exp | 679,318 |
| 25 | 'immunoglobulin replacement':ab,ti,kw | 1,912 |
| 26 | 'immunoglobulin replacement therapy':ab,ti,kw | 1,222 |
| 27 | 'ivig':ab,ti,kw | 26,788 |
| 28 | 'scig':ab,ti,kw | 1,186 |
| 29 | 'subcutaneous immunoglobulin':ab,ti,kw | 1,249 |
| 30 | (immunoglobulin NEAR/3 therapy):ab,ti,kw | 7,210 |
| 31 | (immunoglobulin NEAR/3 therapies):ab,ti,kw | 301 |
| 32 | 'igg rt':ab,ti,kw | 13 |
| 33 | 'igg replacement':ab,ti,kw | 298 |
| 34 | #24 OR #25 OR #26 OR #27 OR #28 OR #29 OR #30 OR #31 OR #32 OR #33 | 685,115 |
| 35 | #6 AND #23 AND #34 | 266 |

Supplementary table 2: CASP checklist for Cohort study with focus on the review question.

| Study | “Teclistamab impairs humoral immunity in patients with heavily pretreated myeloma: importance of immunoglobulin supplementation”(1) | “IVIg use associated with ten-fold reduction of serious infections in multiple myeloma patients treated with Anti-BCMA Bispecific antibodies.” (2) | “Characteristics and incidence of infections in patients with multiple myeloma treated by bispecific antibodies: a national retrospective study.”(3) | “Teclistamab in relapsed refractory multiple myeloma: multi-institutional real-world study.” (4) | “Effect of Intravenous Immunoglobulin (IVIG) Supplementation on infection-free survival in recipients of BCMA-directed bispecific antibody therapy for multiple myeloma”(5) |
| --- | --- | --- | --- | --- | --- |
| **Section A: Are the results valid?** |  |  |  |  |  |
| 1. Did the study address a clearly focused issue? | Yes: “In this study, we aimed to define the impact of Teclistamab on humoral immunity to provide improved guidance on infectious prophylaxis and vaccination strategy.” | Yes: “In this study, we seek to characterize the timing and nature of infections and HGG, elucidate risk factors for infection, and analyze the effect of immunoglobulin replacement on infection rates in patients with multiple myeloma treated with BCMA-directed BiAbs, with the goal of developing rational mitigation strategies for this serious and common toxicity.” | Yes: “We performed a real-world multicenter national study to describe characteristics of infections affecting the management of patients treated with BCMA-targeting or GPRC5D-targeting BsAb and identify their risk factors.” | Yes: …“to assess the efficacy and safety of standard-of-care single-agent teclistamab representing a diverse MM patient population across 5 academic U.S. medical centers.” | Yes: “The main objective of this multi-institutional study is to understand the effect of primary IVIG replacement on clinical outcomes in recipients of BCMA directed bsAb” |
| 1. Was the cohort recruited in an acceptable manner? | Yes, but not reflecting real-world conditions. Inclusion criteria were patients included in MajestTEC trial receiving Teclistamab with stringent inclusion criteria including ECOG 0-1. Patients evaluated for effect of IVIG was from one center. | Yes, but not reflecting real-world conditions. Every patient receiving BCMA-targeted BsAbs was included, all of which was included in clinical studies with probable stringent inclusion criteria. | Yes. All patients receiving BsAbs (both BCMA- and GPRC5D-targeting) during study period were included. It was not specified if these patients were participating in clinical studies. | Yes. Patients receiving Teclistamab during study period at 5 sites. 2 patients had prior BCMA-targeted therapy. Coverage was not reported. | Yes. Patients receiving BCMA-directed bispecific antibodies but including both patients receiving investigational treatment and treatment as standard of care. |
| 1. Was the exposure accurately measured to minimize bias? | No. IVIG was given as either primary or secondary prophylaxis. Of 52 patients receiving teclistamab, 20 received IVIG: 4 was on IVIG prior to start of teclistamab due to history of serious infections, and 16 patients started IVIG early as primary prophylaxis. The remaining 32 patients received IVIG when they experienced severe infection and had polyclonal IgG <4g/L. Patient characteristics and disease related factors were comparable. Other than IVIG, patients received the same microbiological prophylaxis. | No. IVIG was used at the physician’s discretion. 92% of patients received IVIG at some point. Every administration of IVIG was recorded and patients was defined as on IVIG when within 30 days after last administration of IVIG. | No. Exposure to immunoglobulin replacement was not reported. | Yes: 43% received IVIG, all as primary prophylaxis. | No: IVIG was given at the physicians’ discretion, subjecting the results for bias. Patients in the no-IVIG group had higher percentage of investigational BCMA-targeting bispecific antibodies, had higher rates of extramedullary disease and had higher rates of triple class refractory patients compared to the pIVIG group. |
| 1. Was the outcome accurately measured to minimize bias? | Yes, using objective measurements. Outcome was measured as infections microbiologically defined or clinically suspected. Infections were graded by CTCAE version 4.03. | Yes, using objective measurements. Infections were captured by chart review of antibiotic prescription, microbiological data and chart notes, and graded by CTCAE 5.0. | Yes, using objective measurements. Infections were defined clinically, microbiologically or as fever of unknown focus and graded by CTCAE 5,0. Only infections leading to adjustments in treatment or hospitalization was recorded. | Yes, using objective measurements. Infections were defined clinically, microbiologically, histopathologically or by imaging, and graded by CTCAE 5.0. | Yes, using objective measures. Infections were defined clinically, histopathologically, microbiologically and by imaging, and graded by CTCAE 5.0. |
| 1. A) Have the authors identified all important confounding factors? ^[[1]](#footnote-1)^   B) Have they taken account of confounding factors in the design and/or analysis? | A) The authors have identified long term exposure to immunosuppressive therapies before starting BsAb, chronic activation of T-cells with BsAbs, negative effect of BsAbs on normal plasma cells (including reduced vaccine responses) as risks for developing infection other than low immunoglobulins.  Neutropenia during first cycles, T-cell exhaustion and lack of IgA in IVIG was also discussed as possible causes of breakthrough infections. The low incidence of gram-positive infections in the study could be explained by use of antibiotic as primary prophylaxis.  B) In multivariate analyses, adjusting for potential confounding factors was done, without further elaboration. | A) Other than type of study the patient was in, they did not, by univariate regression, identify any risk factors for infection, including steroid use, prior lines of therapy, infections in prior year and high-risk cytogenetics, among others.  B) Excluding the first 30 days of treatment with BsAbs did not impact the effect of IVIG. This was done to limit effect of uncontrolled myeloma, increased health care contact during step-up and CRS-management as confounding factors. | A) The authors identified that corticosteroids for ICANS/CRS were associated with higher risk of first infection, whereas GPRC5D-targeting BsAb and antibiotic prophylaxis was associated with lower risk.  B) Analysis with multivariate adjustment of variables associated with first infection was made. B: Yes. | A) The authors identified that a history of infection was associated with risk of infection. No other cause was associated.  B) Confounding factors was probably not considered. Use of antimicrobials was heterogenous and not considered. | A) Adjusted for immortal time bias.  Multivariate analysis was done to identify confounding factors in estimating progression-free survival and overall survival. The two groups had different number of patients with extramedullary disease and triple-refractory patients. Median number of BsAb doses given and median duration of treatment was also different. |
| 1. A) Was the follow up of subjects complete enough? B) Was the follow up of subjects long enough? | A) Yes. Follow up until end of teclistamab treatment (disease progression or death). Median follow up was 5 months.  B) No. Follow-up was short and there was no follow-up after ending teclistamab treatment. | A) Unclear. Median follow up of 14.1 months. Follow up after end of treatment was not specified.  B) Yes | A) Yes. Infections were reported occurring after completing priming dose and up to 3 months after termination of treatment. Median follow up was 7 months.  B) Yes | A) Unclear. Patients were followed until 60 days after last dose of Teclistamab. Median follow up was 3,5 months.  B) No, follow up was short. | A) Unclear. Follow-up was until end of follow-up or 60 days after last dose of treatment. Median follow-up was 9 months for the pIVIG group and 11 months for the non-pIVIG group. B) Yes |
| **Section B: What was the results?** |  |  |  |  |  |
| 1. What are the results of this study? | Incidence of infections in patients not receiving immunoglobulins was 1.36 per patient year vs 0.12 per patient year in the immunoglobulin group. Cumulative incidence of infections at 6 months: 5.3% with immunoglobulins vs 54.8% with observation only, P < .001. Immunoglobulin replacement was independently associated with decreased risk of severe infection P = .009. | There were 90% fewer grade 3-5 infections in the time on immunoglobulin vs off immunoglobulin, incidence rate ratio 0.10. No significant difference in grade 1-5 infections in patients on immunoglobulin vs off immunoglobulin. | Immunoglobulin substitution was not associated with significant lower risk of infection. Hazard ratio= 0.78. | Cumulative incidence of all grade infections of 35% and 35% at 3 and 6 months respectively in patients on immunoglobulin supplementation, and 44% and 54% in patients off immunoglobulin supplementation, but confidence intervals were overlapping. For grade >2 infections were 17% and 17% in patients on immunoglobulin substitution vs 31% and 43% in patients of immunoglobulin substitution. IVIG as primary prophylaxis was associated with significant lower risk of infections, p = 0,001. | Primary prophylaxis with IVIG is significantly associated with a reduction in infection-free survival and with improved overall survival. Baseline lymphopenia, tocilizumab administration, higher number of median prior lines of therapy and history of prior infection while on bsAb was linked to increased risk of infections. Cumulative infections at 12 months were not significantly different with 56% in the pIVIG group and 60% in the non-pIVIG group. Median infection-free survival was 7,7 months in the pIVIG group and 3 in the non-pIVIG group. Median overall survival was 16 months in the non-pIVIG group and 44 months in the pIVIG group. |
| 1. How precise are the results? | The study reports on type of infections, but the exposure in the two groups is not consistent, as 14 of 18 patients cross over from non-IVIG (observational group) to IVIG. This study is non-randomized and retrospective with the potential for selection bias. The results are evaluated as somewhat precise. | Exposure was not consistent as IVIG was given at physician’s discretion. Outcome was accurately measured. This study is non-randomized and retrospective with the potential for selection bias. Many patients received suboptimal doses of BsAbs and some had received prior non-BCMA antibodies.  The results are evaluated as somewhat precise. | Not clear. Immunoglobulin substitution was not significantly associated with lower risk of infections, but exposure to immunoglobulin substitution was not specified. Outcome was accurately measured. This study is non-randomized and retrospective with the potential for selection bias. It was also heterogenous in prophylactic treatment and type of BsAb. The results are evaluated as less precise. | The exposure was clear, and the outcome was accurately measured. The study had heterogenous use of antimicrobial prophylaxis. The study is non-randomized and retrospective with the potential for selection bias. The results are evaluated as somewhat precise. | The exposure was clear, but IVIG was given at the physician’s discretion, subjecting the results for bias. There was also a difference in the disease burden, refractoriness to earlier treatment and duration and doses of bsAb treatment in the two groups compared. The study is non-randomized and retrospective with the potential for selection bias. The results are evaluated as somewhat precise. |
| 1. Do you believe the results? | Yes | Yes. | Unclear | Yes | Yes |
| **Section C: Will these results help locally?** |  |  |  |  |  |
| 1. Can the results be applied to the local population? | As inclusion was based on stringent inclusion criteria the results do not reflect a true real-world population. | It’s a small study including 37 patients from different clinical trials, and do not reflect a true real-world population. | This is a large real-world study applicable to the local population. However, the results regarding effect of immunoglobulin substitution are unclear as the exposure is not described in the article. | This large real-world study including ethnic minorities are applicable to the local population. | Yes, for the teclistamab standard-of-care group. Some of the patients was receiving investigational treatment and was probably subjected to stringent inclusion criteria making the results less applicable to the local population. |
| 1. Do the results of this study fit with other available evidence? | Yes | Yes | No | Yes | Yes |
| 1. What are the implications of this study for practice? | It implies the need for prophylactic immunoglobulin treatment during teclistamab treatment. | It implies the need for prophylactic immunoglobulin treatment during BsAb treatment. | The high rates of bacterial infections may support the role of IVIG supplementation. | It implies the need for prophylactic immunoglobulin treatment during BsAb treatment. | It implies that the use of immunoglobulin substitution may influence the infection free survival and may improve the overall survival of the patients. |

BsAbs/BiAbs: Bispecific antibodies, MM: Multiple Myeloma, IVIG: Intravenous immunoglobulins, ECOG: eastern cooperative oncology group (performance status scale), HGG: hypogammaglobulinemia, CTCAE: Common terminology criteria for adverse events, BCMA: B-cell maturation antigen GPRC5D: G-coupled protein receptor, class C, group 5, member D, pIVIG primary intravenous immunoglobulin prophylaxis.

Available from: <https://casp-uk.net/casp-tools-checklists/cohort-study-checklist/>

1. Frerichs KA, Verkleij CPM, Mateos MV, Martin TG, Rodriguez C, Nooka A, et al. Teclistamab impairs humoral immunity in patients with heavily pretreated myeloma: importance of immunoglobulin supplementation. Blood Adv. 2024;8(1):194-206.

2. Lancman G, Parsa K, Kotlarz K, Avery L, Lurie A, Lieberman-Cribbin A, et al. IVIg Use Associated with Ten-Fold Reduction of Serious Infections in Multiple Myeloma Patients Treated with Anti-BCMA Bispecific Antibodies. Blood Cancer Discov. 2023;4(6):440-51.

3. Jourdes A, Cellerin E, Touzeau C, Harel S, Denis B, Escure G, et al. Characteristics and incidence of infections in patients with multiple myeloma treated by bispecific antibodies: a national retrospective study. Clinical Microbiology and Infection. 2024;30(6):764-71.

4. Mohan M, Monge J, Shah N, Luan D, Forsberg M, Bhatlapenumarthi V, et al. Teclistamab in relapsed refractory multiple myeloma: multi-institutional real-world study. Blood Cancer J. 2024;14(1):35.

5. Mohan M, Szabo A, Cheruvalath H, Clennon A, Bhatlapenumarthi V, Patwari A, et al. Effect of Intravenous Immunoglobulin (IVIG) Supplementation on infection-free survival in recipients of BCMA-directed bispecific antibody therapy for multiple myeloma. Blood Cancer J. 2025;15(1):74.

1. Possible confounding factors are factors leading to infection or discovery of an infection and that might lead to administration of immunoglobulin substitution. Possible factors are many including age of patient, line of therapy, earlier treatment (autologous bone marrow transplant, steroids, immunomdulatory agents etc), infectious prophylaxis, frequent hospital visits at treatment start or in clinical trials, B- and T-cell numbers, neutropenia, vaccination status, hypogammaglobulinemia, comorbidity etc. [↑](#footnote-ref-1)
